# Increased risk and related factors of depression among patients with COPD: a population-based cohort study

**DOI:** 10.1186/1471-2458-13-976

**Published:** 2013-10-19

**Authors:** Tzung-Yi Tsai, Hanoch Livneh, Ming-Chi Lu, Pang-Yau Tsai, Pei-Chun Chen, Fung-Chang Sung

**Affiliations:** 1 Department of Medical Research, Buddhist Dalin Tzu Chi Hospital, Chiayi, Taiwan; 2 Department of Public Health, China Medical University, Taichung, Taiwan; 3 Department of Nursing, Tzu Chi College of Technology, Hualien, Taiwan; 4 Department of Special Education and Counselor Education, Portland State University, Portland, Oregon, USA; 5 Division of Allergy, Immunology and Rheumatology, Buddhist Dalin Tzu Chi Hospital, Chiayi, Taiwan; 6 School of Medicine, Tzu Chi University, Hualien, Taiwan; 7 Management Office for Health Data, China Medical University Hospital, Taichung, Taiwan; 8 Institute of Epidemiology and Preventive Medicine, National Taiwan University College of Public Health, Taipei, Taiwan

**Keywords:** Chronic obstructive pulmonary disease, Depression, Cohort study

## Abstract

**Background:**

Depression is a common and mostly undertreated problem in patients with chronic diseases. However, population-based studies on the association between chronic obstructive pulmonary disease (COPD) and subsequent depression are limited in Asian populations. This study evaluated the incidence and risk factors of depression for patients with COPD in Taiwan.

**Methods:**

Using the claims data from the National Health Insurance of Taiwan, we identified 38,010 COPD patients newly diagnosed in 2000–2004 and 38,010 subjects without COPD frequency, matched by sex, age and index date. The incidence rate and hazard ratio for depression were estimated by the end of 2008.

**Results:**

The incidence rate of depression was 1.88 folds higher in the COPD cohort than in the non-COPD cohort (12.2 versus 6.47 per 1,000 person-years, p < 0.0001). The depression risk was the greatest within the first year following COPD diagnosis and tended to decline with follow-up time. Among COPD patients, multivariate analysis showed that younger women and low-income patients were at higher risk of depression. Hospitalization and comorbidities such as hypertension, arthritis, cancer, and heart disease were also significant predictors for depression risk.

**Conclusion:**

This population-based cohort study demonstrated a strong relationship between COPD and subsequent depression. These findings could assist healthcare providers to pinpoint individuals with a higher predisposition to having depression, which could then facilitate the provision of culturally appropriate rehabilitation within the first year after the diagnosis of COPD.

## Background

Chronic obstructive pulmonary disease (COPD) is characterized by airflow obstruction, which leads to the slow, progressive symptoms of persistent cough, exertional dyspnea, wheezing, and eventually functional impairment [1]. World Health Organization (WHO) has reported in the Global Burden of Disease Project that approximately 5 of 100 deaths worldwide are associated with COPD [2]. At this rate, COPD may rank as the third leading cause of death in the world by 2020 [3]. Given the complex symptoms and long duration of COPD, the medical expenses for curing the disease cannot be ignored. A study by Menzin et al. [4] found that the average medical expenses per COPD patient per year was estimated at US$27,656, which is nearly 4 times higher than the average cost for patients without COPD (US$7,126). In a review of the financial burden of COPD in USA, the authors estimated that the annual direct medical costs for COPD amounted to $21.8 billion, and the indirect costs for restricted days, lost workdays, and productivity were at $17 billion [5].

COPD does not only cause enormous economic burden, it also triggers subsequent illnesses. The risk of lung cancer for COPD patients is more than two folds higher than for those without the disease [6]. Curkendall et al. also confirmed that COPD patients have a 60%–80% higher risk of developing cardiovascular diseases compared with the general population [7]. Given the irreversible nature and unsatisfactory prognostic outcomes, COPD patients are frequently diagnosed with psychiatric disorders, particularly depression [8]. A systematic review of 64 studies on patients with chronic disease concluded that the prevalence of depression ranged from 37% to 71% in COPD patients, figures comparable to or higher than prevalence rate in patients with other chronic diseases, such as cancer, AIDS, heart disease, and renal disease [9]. Depression not only increases hospitalization and emergency visits by 48% and 77%, respectively, in COPD patients [10], but also doubles the likelihood of dying from the comorbidities of COPD-related depression [11]. Therefore, depression has become a matter of great healthcare concern for COPD patients and their families.

Studies on COPD-related depression have been conducted mainly on Western populations but rarely for people of Asian descent [12]. Chinese, in particular, often consider depression to be a taboo issue and they may suffer more in terms of emotional distress [13,14]. Previous studies on Chinese COPD patients have focused on the effect of medical treatments [15], mortality [16], and the subsequent risk of skin disease following COPD [17]. In contrast, data related to the psychological issues, especially depression, among Asian patients with COPD are limited. Few studies investigating the influencing factor of depression among Chinese COPD patients have been conducted, but these studies were limited to a cross-sectional design and small sample sizes [18,19]. We conducted a follow-up study to determine the association between depression risk and COPD using claims data from the National Health Insurance (NHI) of Taiwan to evaluate the risk of depression in Oriental patients with COPD. Apart from being a preliminary study of depression in COPD patients, its findings should be able to help healthcare providers identify potential cases of having depression and this will allow more efficient interventions to improve the quality of life for them.

## Methods

### Data source

In order to remove financial barriers to medical care for all residents, Taiwan Department of Health launched a single-payer NHI Program in 1995. At the end of 2008, over 99% of Taiwan’s population was enrolled in this program [20]. The National Health Research Institutes in Taiwan has been responsible to manage the insurance claims data and established several sets of Longitudinal Health Insurance Databases (LHID), providing to scientists in Taiwan for research purposes. This study used a subset of NHI database, which contained the utilization and enrollment information for a randomly selected of one million NHI beneficiaries, representing 5% of all enrollees in Taiwan in 2000. Because a multistage stratified systematic sampling method was applied, there were no statistically significantly differences in sex or age between the sample group and all enrollees [20]. All data files can be linked with scrambled identifications. Our study was thus exempted from full review by the Institutional Review Board (Research Ethnics Committee, Buddhist Dalin Tzu Chi Hospital).

### Study population

Diagnoses in the insurance claims data were coded with International Classification of Disease, 9th Revision, Clinical Modification (ICD-9-CM). From records in LHID, we identified patients with COPD among adults newly diagnosed during 2000–2004 with the ICD codes of 491, 492, and 496 as the COPD cohort. To reduce the misclassification, we selected patients with at least two diagnosis of COPD in outpatient visits within 12 months or patients being admitted to hospital with a primary diagnosis of COPD during the 5-year period [17,21].

A comparison cohort was randomly selected from the remaining insured population without COPD. For each COPD patient, one person free of COPD was selected frequency matched with sex, age, and the index date of COPD. After excluding subjects with a history of depression at the baseline, we identified 38,010 patients with COPD and 38,010 subjects in non-COPD cohort for data analysis. All subjects were followed up to the end of 2008 to measure the incidence of depression. We identified subjects as having depression if they had at least two treatment claims for depression in outpatient visits or hospitalization with depression for ICD-9-CM codes of 296.2, 296.3, 300.4, or 311 during the follow-up period. Follow-up person-years (PYs) were determined by calculating the time interval from the entry date to the earliest of one of the following: a diagnosis of depression, the date of withdrawal from the insurance, or the date of December 31, 2008.

### Demographic variables and comorbidities

The demographic variables used in this study included age, gender, income for estimating insurance payment, and urbanization of the subject’s residential area. The monthly incomes were divided into three levels: ≤NT$17,880, NT$17,881–$43,900, and ≥NT$43,901. An income of NT$17,880 was the government-stipulated minimum for full-time employees in Taiwan. Urbanization levels were stratified into three strata, namely urban (levels 1–2), suburban (levels 3–4) and rural (levels 5–7) areas, based on population density. Level 1 refers to the “most urbanized” and level 7 refers to the “least urbanized” communities [22]. The baseline comorbidity was also identified for each subject, including hypertension (ICD9-CM 401–405), arthritis (ICD9-CM 715, 716.90), diabetes (ICD9-CM 250), heart disease (ICD9-CM 410–429), chronic kidney disease (ICD9-CM 585), and cancer (ICD9-CM 140–208).

### Statistical analysis

We performed the χ^2^ test to examine the differences in demographic characteristics and comorbidities between the COPD and comparison cohorts. The incidence rate of depression for the two cohorts was presented with the number of cases per 1,000 PYs. COPD patients were further divided into two groups based on initial way of medical care seeking, and examined the incidence rate of depression among them. Cox proportional hazards regression analysis was applied to compute the hazard ratio (HR) and 95% confidence intervals (CI) of depression for COPD compared with the comparison cohort. We also used multivariate Cox proportional hazards model to determine the risk factors that might predict depression and their adjusted hazard ratio (aHR) within the COPD cohort. We further evaluated whether the relation between COPD and depression risk differed over time by sex. All analyses were conducted using SAS version 9.3 (SAS Institute Inc., Cary, NC, USA), and p < 0.05 was considered significant.

## Results

### Baseline and depression incidence between two cohorts

Table 1 shows the distributions of the demographic data and comorbid medical disorders for the COPD and comparison cohorts. Patients with COPD were more likely to have a lower monthly income (p < 0.0001), reside in rural areas (p < 0.0001), and suffer comorbidities, such as hypertension, arthritis, diabetes, heart disease, chronic kidney disease, and cancer (all p < 0.0001).

**Table 1:** Demographic status and comorbidity compared between cohorts with and without COPD

**Variables**	**Non-COPD**	**COPD**	***p***
	**N = 38010**	**N = 38010**	
Age, years	n (%)	n (%)	
<50	11209 (29.5)	11097 (29.2)	0.3245
50–69	14515 (38.2)	14436 (38.0)	
≥70	12286 (32.3)	12477 (32.8)	
mean (SD)	59.0 (17.1)	59.2 (17.0)	0.1365
Gender			1.0000
Female	16344 (43.0)	16344 (43.0)	
Male	21666 (57.0)	21666 (57.0)	
Monthly income			<0.0001
Low	16787 (44.2)	16338 (43.0)	
Median	19109 (50.3)	19757 (52.0)	
High	2114 (5.60)	1915 (5.00)	
Level of urbanization			<0.0001
Urban	21149 (55.9)	20479 (54.1)	
Suburban	12327 (32.6)	12465 (32.9)	
Rural	4373 (11.6)	4919 (13.0)	
Comorbidity			
Hypertension	10565 (27.8)	15190 (40.0)	<0.0001
Arthritis	6390 (16.8)	9799 (25.8)	<0.0001
Diabetes	4474 (11.8)	6027 (15.9)	<0.0001
Heart disease	6473 (17.0)	11871 (31.2)	<0.0001
Chronic kidney disease	601 (1.60)	903 (2.40)	<0.0001
Cancer	1591 (4.20)	2480 (6.50)	<0.0001

Of the total sample of 76,020 patients, 4,109 had incident depression during the follow-up period. The incidence of depression in the COPD cohort was nearly two folds higher than that in the comparisons (12.19 vs. 6.47 per 1,000 PYs) with an aHR of 1.88 (95% CI 1.74–1.98; Table 2). The depression risk was slightly greater for inpatients than for outpatients with COPD, with aHR of 2.29 (95% CI: 1.98–2.65) and 1.85 (95% CI: 1.74–1.98).

**Table 2:** Crude and adjusted hazard ratios of depression for outpatients and inpatients of COPD compared with non-COPD controls

**Patient group**	**Event**	**PY**	**Incidence**	**Crude HR**	aHR*
				**(95% CI)**	**(95% CI)**
Controls	1477	228176	6.47	1.00	1.00
COPD patients	2632	215852	12.19	1.88 (1.76–2.00)	1.88 (1.77–2.01)
COPD outpatient	2418	202437	11.94	1.85 (1.73–1.97)	1.85 (1.74–1.98)
COPD inpatient	214	13415	15.95	2.40 (2.08–2.77)	2.29 (1.98–2.65)

Per 1,000 person-years for incidence rate.

*Model adjusted for age, sex, urbanization level, income, and the comorbidity.

### Relative risk of depression among COPD cohorts

The multivariate Cox proportional-hazard regression analysis estimated the aHR of depression in COPD patients by demographic status and comorbidity (Table 3). Age, gender, monthly income and hospitalization were significantly related to the risk of depression in COPD patients. Compared with the oldest group (≥70 years old), the risk of depression was 15% higher for those aged ≦ 50 years of age; those aged 51–70 also had an excess risk of 11%. Female sex was related to an aHR of 1.19 for depression, as compared to male. With regard to monthly income, aHR compared to the subjects with high monthly income was 1.09 for those with median monthly income, and 1.23 for those with low monthly income. Furthermore, the aHR was 1.16 times greater for COPD inpatients than COPD outpatients. Comorbidity increased the depression risk, with the highest risk for those with heart disease (aHR 1.47, 95% CI 1.35–1.61), followed by those with arthritis (aHR 1.32, 95% CI 1.21–1.44), cancer (aHR 1.24, 95% CI 1.06–1.45), and hypertension (aHR 1.12, 95% CI 1.03–1.23).

**Table 3:** Multivariate Cox proportional hazards regression analysis measures the risk of depression among COPD patients

**Variable**	**Number of depression**	**Incidence per 1,000 person-years**	**Crude HR**	aHR*
			**(95% CI)**	**(95% CI)**
Age				
<50	770	10.91	0.80 (0.72–0.88)	1.15 (1.02–1.28)
50–69	1043	12.01	0.91 (0.83–1.00)	1.11 (1.01–1.23)
≥70	819	14.02	1.00	1.00
Gender				
Female	1305	13.64	1.23 (1.15–1.33)	1.19 (1.10–1.28)
Male	1327	11.04	1.00	1.00
Monthly income				
Low	1189	13.14	1.41 (1.16–1.72)	1.23 (1.01–1.67)
Median	1333	11.75	1.11 (1.03–1.20)	1.09 (1.00–1.18)
High	110	9.25	1.00	1.00
Level of urbanization				
Urban	1473	12.34	1.00	1.00
Suburban	821	11.80	0.95 (0.88–1.04)	0.95 (0.87–1.04)
Rural	328	12.60	1.01 (0.90–1.14)	0.98 (0.87–1.11)
Hospitalization				
Outpatients	2418	11.94	1.00	1.00
Inpatient	214	15.95	1.29 (1.12–1.48)	1.16 (1.01–1.34)
Comorbidity				
Hypertension				
No	1470	10.72	1.00	1.00
Yes	1162	14.76	1.35 (1.25–1.46)	1.12 (1.03–1.23)
Arthritis				
No	1795	10.91	1.00	1.00
Yes	837	16.33	1.47 (1.36–1.60)	1.32 (1.21–1.44)
Diabetes				
No	2180	11.71	1.00	1.00
Yes	452	15.24	1.27 (1.15–1.41)	1.10 (0.99–1.23)
Heart disease				
No	1619	10.35	1.00	1.00
Yes	1013	17.03	1.61 (1.49–1.75)	1.47 (1.35–1.61)
Chronic kidney disease				
No	2571	12.10	1.00	1.00
Yes	61	17.75	1.41 (1.09–1.81)	1.14 (0.88–1.47)
Cancer				
No	2462	11.98	1.00	1.00
Yes	170	16.54	1.34 (1.15–1.56)	1.24 (1.06–1.45)

*Adjusted for all variables in the model.

Additionally, we found the risk of depression across the period following COPD diagnosis was 2.01 (95% CI: 1.72–2.32) in year one. The aHR decreased with follow-up time to 1.40 (95% CI: 1.22–1.60) after year 5 (Table 4). The sex-specific patterns during the follow-up period were similar for males and females, but higher for females.

**Table 4:** Sex-specific hazard ratios of depression in COPD patients relative to non-COPD controls by follow-up duration

**Follow-up duration**	**Comparison**		**COPD**		aHR^*^
	**Event**	**Rate**	**Event**	**Rate**	**(95% CI)**
Total					
Year <1	262	7.02	607	16.68	2.01 (1.72-2.32)
Year 1–3	484	6.83	842	12.55	1.66 (1.48-1.87)
Year 3–5	373	5.26	662	9.97	1.65 (1.45-1.89)
After year 5	358	6.37	521	9.81	1.40 (1.22-1.60)
Female					
Year <1	112	5.27	318	15.44	2.44 (1.96-3.04)
Year 1–3	220	5.48	433	11.53	1.92 (1.62-2.26)
Year 3–5	193	6.26	316	8.55	1.63 (1.35-1.97)
After year 5	149	4.73	260	8.91	1.70 (1.38-2.09)
Male					
Year <1	150	9.32	289	18.30	1.86 (1.40-2.19)
Year 1–3	264	8.61	409	13.86	1.67 (1.36-2.04)
Year 3–5	180	4.49	346	11.76	1.45 (1.24-1.71)
After year 5	209	8.46	261	10.90	1.17 (0.97-1.42)

^*^Adjusted for age, sex, urbanization level, income, and comorbidity.

## Discussion

Previous studies have shown that emotional impairments, depression in particular, are common among COPD patients, but mainly in Western populations. To the best of our knowledge, the present study is the first population-based cohort study addressing the association between COPD and the risk of subsequent depression in an Asian population. The results are consistent with findings in previous studies on Western populations [8,23,24]. The likely explanation for this finding was as follows. COPD patients may require more daily care due to gradually worsening physical conditions, which may lower self-esteem and self-efficacy, inducing a higher risk of depression [9]. Schane et al. found that COPD patients of lower education, living alone and female gender were more likely to have depression [8]. A cross-sectional study in China also found low income COPD patients are more likely depressive [25]. Our study revealed similar socioeconomic association, indicating the elderly and low income COPD patients are at higher risk of depression.

Additionally, the same debilitating and continuous physical symptoms may also trigger cognitions of “learned helplessness”, such that one’s inability to successfully cope with physical deterioration and life threatening situations result in increased levels of uncertainty, unpredictability and ultimately emotional distress and depression [26]. On the other hand, several inflammatory cytokines, such as tumor necrosis factor-α (TNF-α) or interleukin-6 (IL-6), have been considered pathogenic factors associated with the mechanism of developing depression [27]. A meta-analysis containing 24 studies indicated that the inflammatory cytokines may regulate adult neurogenesis to induce hippocampal neurogenesis atrophy, which has been implicated as a key contributing mechanism in the pathophysiology and treatment of depression [28].

The depression incidence among COPD patients in this study is somewhat lower than that in a previous report [24]. The difference may stem from the different composition of the two groups of participants. In addition, the stigma of mental illness is particularly entrenched in Asian patients and they may be unwilling to acknowledge or admit feelings of depression [13]. Depression, in many Asian countries, is an embarrassing issue for the general population to openly seek regular psychotherapy. Most importantly, less than 20% of patients with COPD have ever received appropriate psychiatric treatment since they were diagnosed with COPD [19]. Therefore, the implementation of a standardized care process, including some screening tools applicable to COPD patients, may be of utmost importance.

Our study indicated that women are at a 1.19-fold greater risk of depression than men in the COPD cohort, which supports the findings of previous studies [8,24]. Two reasons may account for this phenomenon. First, women are more health consciousness than men and more likely to seek medical care during irregularities in their well-being [29]. Moreover, women are traditionally more obedient, passive, and emotional, which may insidiously elevate the risk of depression [30]. In contrast, our finding contradicts those reported by van Manen et al. [31]. A small sample size of 162 subjects used in their study may lead to a null association between the depression risk and gender.

We found a lower risk of depression in patients with higher monthly incomes than in those with lower monthly incomes, which is in line with previous studies [30,32]. Patients with higher incomes may have better financial resources in dealing with the challenges engendered by COPD. This result, however, is inconsistent with that reported by Lin et al. [33], which may stem from the use of a different study design. Given that establishing a clear causality with the cross-sectional design is difficult, erroneously determining the effect of risk factors is possible [34].

Consistent with previous studies [8,18,31], we found that younger COPD patients were at a higher hazard for depression than the elderly. Most of the young COPD patients were employed; therefore their workload may have contributed to additional emotional distress. More importantly, our results demonstrated that the incidence of depression is higher in COPD inpatients than in outpatients. This finding indicates that severe COPD, which requires hospitalization, may trigger an increased risk of depression. Similar to previous report [35], we also noted that the risk of depression in COPD patients was higher within one year after diagnosis. The newly diagnosed patients may be highly fragile and overwhelmed by COPD symptoms. These findings suggest that healthcare providers need to pay closer attention to newly diagnosed patients with COPD, particularly inpatients. Interventions geared toward providing emotional support to enhance the psychological adjustment of patients may be of utmost importance.

COPD patients with other comorbidities, such as hypertension, arthritis, heart disease, or cancer had a significantly higher risk of depression, whereas those with diabetes mellitus and chronic kidney disease were at borderline significance. Our findings are partially paralleled with those of a previous study [8], indicating that comorbidities are associated with the predisposition for depression among COPD patients. The occurrence of comorbidities could play a role in weakening the perceptions of health status among COPD patients, thus leading to increased risk of depression. Additionally, a review article demonstrated that a depressive mood might be related to medication, such as antihypertensive agents, corticosteroids, or other biological agents [36]. So it was suggested that clinicians should actively assess existing medications prior to initiating depression-related treatments for COPD patients, which may reduce the risk of depression after COPD diagnosis.

Several limitations in this study should be mentioned. First, information on tobacco use, social network relationships, religious belief, and education level was unavailable from the claims data, which may be related to depression. Further study linking administrative data and the above mentioned information is warranted to examine the impact of these potential confounding variables on depression. Second, inaccurate diagnoses may occur. Therefore, we selected only those subjects with COPD or depression with at least two consistent outpatient visits or one inpatient admission to minimize this error. Furthermore, NHI has randomly sampled claims from hospitals and randomly interviewed patients, as well as reviewed medical charts to verify the accuracy of medical records. Finally, data regarding the severity of COPD, such as lung function data, were not available in the database. We were unable to estimate the dose–response relations between COPD severity and depression. Despite these methodological concerns, this population-based cohort study has been successful in examining the relationship between COPD and the subsequent risk of depression among Chinese patients. The retrospective cohort study design per se provided stronger evidence on the relationship between COPD and depression risk compared with previous studies.

## Conclusions

Findings from this study reveal that the depression risk is nearly two folds higher for COPD patients than for general population. The risk of depression is related to being female and young, as well as with lower monthly income and comorbidity, such as hypertension, arthritis, cancer, and heart disease. Healthcare providers could be able to better understand the demographic and diseases characteristics that may contribute to the depression risk from this population-based study. The need to routinely screen COPD patients for depression and institute culturally appropriate interventions should be emphasized, especially within the first year since COPD diagnosis. Healthcare providers must be cognizant of the existence of depression among COPD patients and inform them by standard care process to avoid potential embarrassment when discussing psychiatric-related issues; this will help to achieve better therapeutic outcomes for them.

## Competing interests

The authors declare that they have no competing interests.

## Authors’ contributions

Conceived and designed the experiments: TYT HL MCL FCS PCC. Performed the experiments: TYT FCS PCC. Analyzed the data: TYT PYT. Wrote the paper: TYT FCS PCC. All authors approved the final version of the manuscript. PCC is the co-corresponding author.

## Pre-publication history

The pre-publication history for this paper can be accessed here:

http://www.biomedcentral.com/1471-2458/13/976/prepub
